# Tissue expansion for challenging DBS hardware erosions in patients with Parkinson's disease

**DOI:** 10.1016/j.bas.2022.101188

**Published:** 2022-09-16

**Authors:** Nikhil Thakur, Michael Eibach, Shahram Ghanaati, Lutz Weise, Volker Seifert, Gerhard Marquardt, Johanna Quick-Weller

**Affiliations:** a Department of Neurosurgery, Johann Wolfgang Goethe University Hospital, Frankfurt, Germany; b Department of Oral- Cranio- Maxillofacial and Facial Plastic Surgery, Johann Wolfgang Goethe University Hospital, Frankfurt, Germany; c Division of Neurosurgery, Dalhousie University, Halifax, Canada

**Keywords:** Deep brain stimulation, Parkinson's disease, Tissue expander, Impulse generator, Scalp defect, Hardware erosion

## Abstract

• Consider tissue expanders for challenging DBS cases in PD patients with hardware erosion.
• Placement of tissue expander is essential in planning the reconstruction.
• MRI-compatibility of the tissue expander is paramount for shortening the total duration of anesthesia.
• Role of routine skin biopsies to identify PD patients at additional risk for developing scalp defects should be investigated.

## 1 Introduction

Scalp defects (SD) after neurosurgical procedures tend to be challenging due to the potential of developing infections in the underlying tissue. This problem is greatly aggravated when meticulously planned – not to mention, expensive, implanted electrical leads are present underneath the SD. (Sixel-Döring et al., 2010)

High-volume DBS centers are inevitably faced with challenging cases, where surgical debridement alongside secondary adaptation and closure does not suffice. Parkinson's Disease (PD) is a multi-systemic nervous system disorder with studies indicating an increased possibility of a disease-related peripheral neurodegeneration when findings of alpha-synuclein deposits in skin nerve fibers are present (Dabby et al., 2006). PD patients have a reduced density of small intraepidermal nerve-fibers. Additionally, the innervation of sweat gland and erector pili muscles is dwindled down (Wang et al., 2013; Nolano et al., 2008). According to Doppler et al. there is a highly significant difference between deposits of phosphorylated alpha-synuclein in PD patients and healthy controls (Doppler et al., 2014).

Methods to preemptively deal with looming hardware infections due to skin erosion in a patient group that is plagued by autonomic dysregulation (Dabby et al., 2006; Wang et al., 2013; Nolano et al., 2008; Doppler et al., 2014; Skorvanek and Bhatia, 2017) and possibly insufficient nutrition (Ehlen et al., 2013; C et al., 2013; Mancini et al., 2014) by using an acellular dermal matrix have been described in the literature (Barrett et al., 2018). While preemptive surgery for impending skin erosions might offer an alternative in some cases, many patients in long-term follow-up might present with an already significant SD or worse still, with contaminated implants and signs of a systemic infection.

Tissue expanders (TE) have been used in plastic and reconstructive surgery for over half a century (Neumann, 1957) in a myriad of pathologies. Usual/contemporary utilization of TEs is for post traumatic, breast and other aesthetic reconstructions. The scanty neurosurgical literature on this topic deals with reconstructive procedures after multiple craniotomies for neuro-oncological and neurotrauma cases, for complicated cranioplasties (Carloni et al., 2015, 2016; Merlino and Carlucci, 2015) and more recently for a neurovascular case (Jayapaul et al., 2018) The use of this technique for complicated DBS patients has not yet been reported. We discuss the considerations in opting for this procedure, challenges in selecting the right implants and timing of the surgical interventions. An illustrative case of a 60-year-old male DBS patient with PD treated with this technique is discussed in some detail.

## 2 Method

**Image1:**
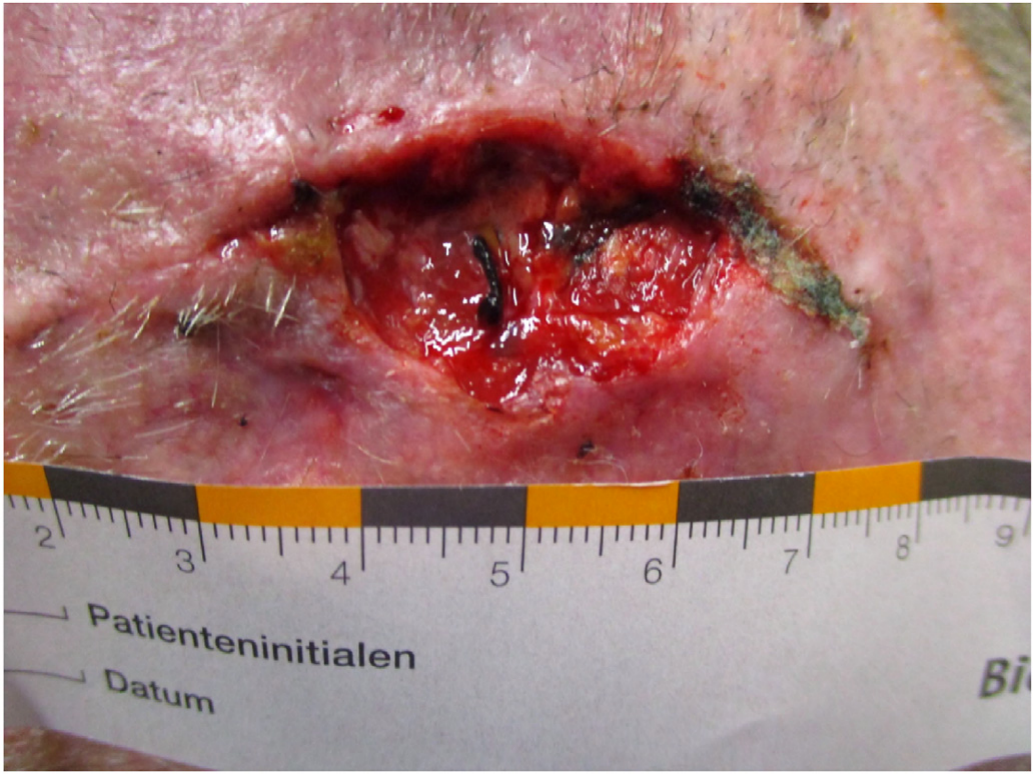
Scalp Defect with underlying DBS leads.

The patient had undergone DBS surgery with bilateral STN stimulation for idiopathic PD. A total of 6 revision procedures had to be carried out due to infections both at the site of the implanted impulse generator (IPG) and at the level of the scalp directly above the DBS electrodes/Burr-Hole covers. The IPG and eventually the DBS electrodes had to be explanted due to bacterial implant contamination with an open frontal scalp defect (Image1). Since the patient fared poorly without DBS with a substantial worsening of his parkinsonian symptoms and thus, quality of life (QoL), another DBS procedure after sufficient wound-healing was deemed necessary.

**Image2:**
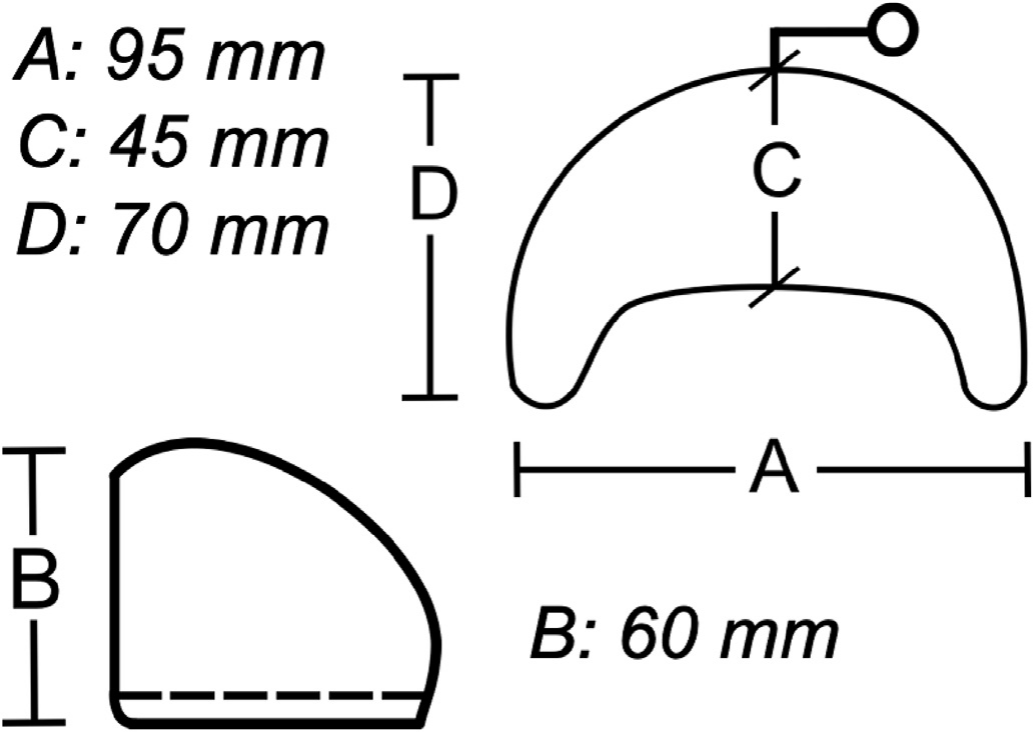
Crescent shaped Polytech POLYsmoooth™ 200 ​ml tissue expander with a 17 ​mm remote valve. Illustration taken and adapted from the Polytech product brochure with A: 95 ​mm, B: 60 ​mm, C: 45 ​mm and D: 70 ​mm.

In accordance with an interdisciplinary consensus between the departments of Neurosurgery, Infectious Diseases and Oral- Cranio- Maxillofacial & Facial Plastic Surgery (MFPS), occipital implantation of a crescent shaped Polytech POLYsmoooth™ 200 ​ml tissue expander with a 17 ​mm remote valve (Image2) was carried out during the surgical removal of the contaminated implants in addition to postoperative antibiotic-therapy with amoxicillin/clavulanic acid.

Since we used the Leksell coordinate frame G, attention was given to leave ample room to place pins for lateral mounting following the centre of the arc principle (Zrinzo, 2012), without puncturing or hindering the removal of the TE (Illustration 1). Close communication with the oral-cranio-maxillofacial surgeon at this planning step was a priority. A member of our neurosurgical team was present during the implantation of the TE. Specific attention was given to highest possible MRI-compatibility and placement of the TE for the purposes of the eventual re-implantation. The manufacturer of the TE was contacted to ascertain the materials utilized and the method used in production. External MRI Safety data was unfortunately not available.

**Image3:**
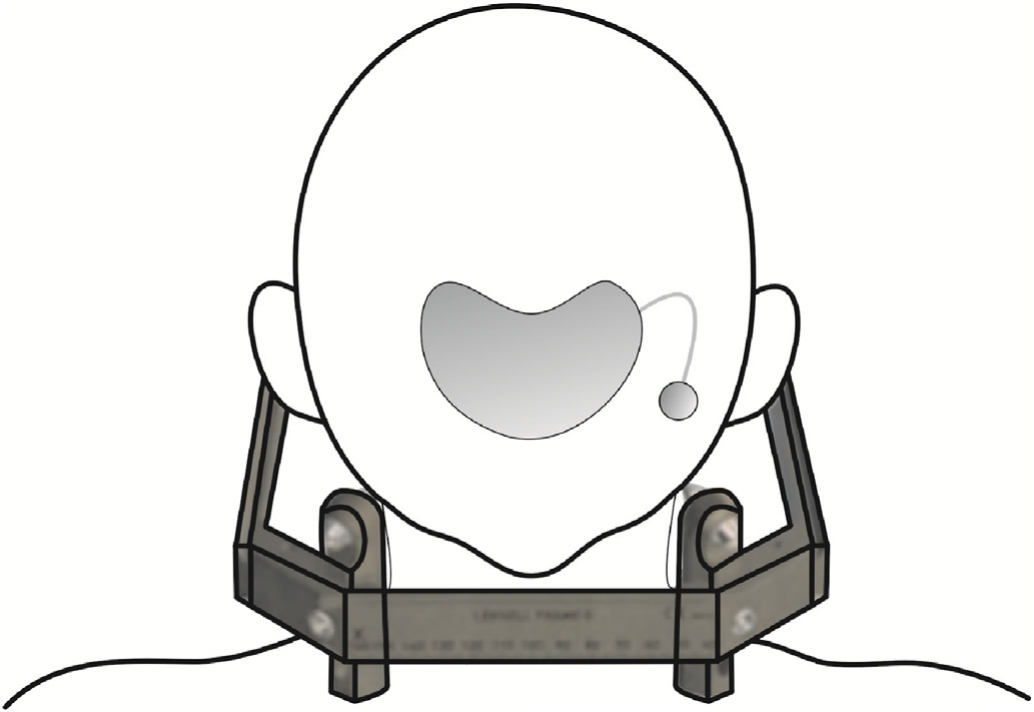
Importance of TE-positioning and MRI-compatibility.

The tissue expansion was then gradually achieved in 10 ​ml steps over a period of 7 months (Image3) at the discretion of MFPS based on skin-elasticity. Additional wound documentation and care was provided by the wound center at our university hospital in an ambulatory setting. Regular blood sampling was conducted to ensure that no further systemic infection flared up. After sufficient expansion and ensuring that the patient had no signs of local or systemic infection, a second DBS procedure was planned. Painstaking precautions were taken during the MRI to avoid any adverse events because of missing external MRI Safety information.

**Illustration 1:**
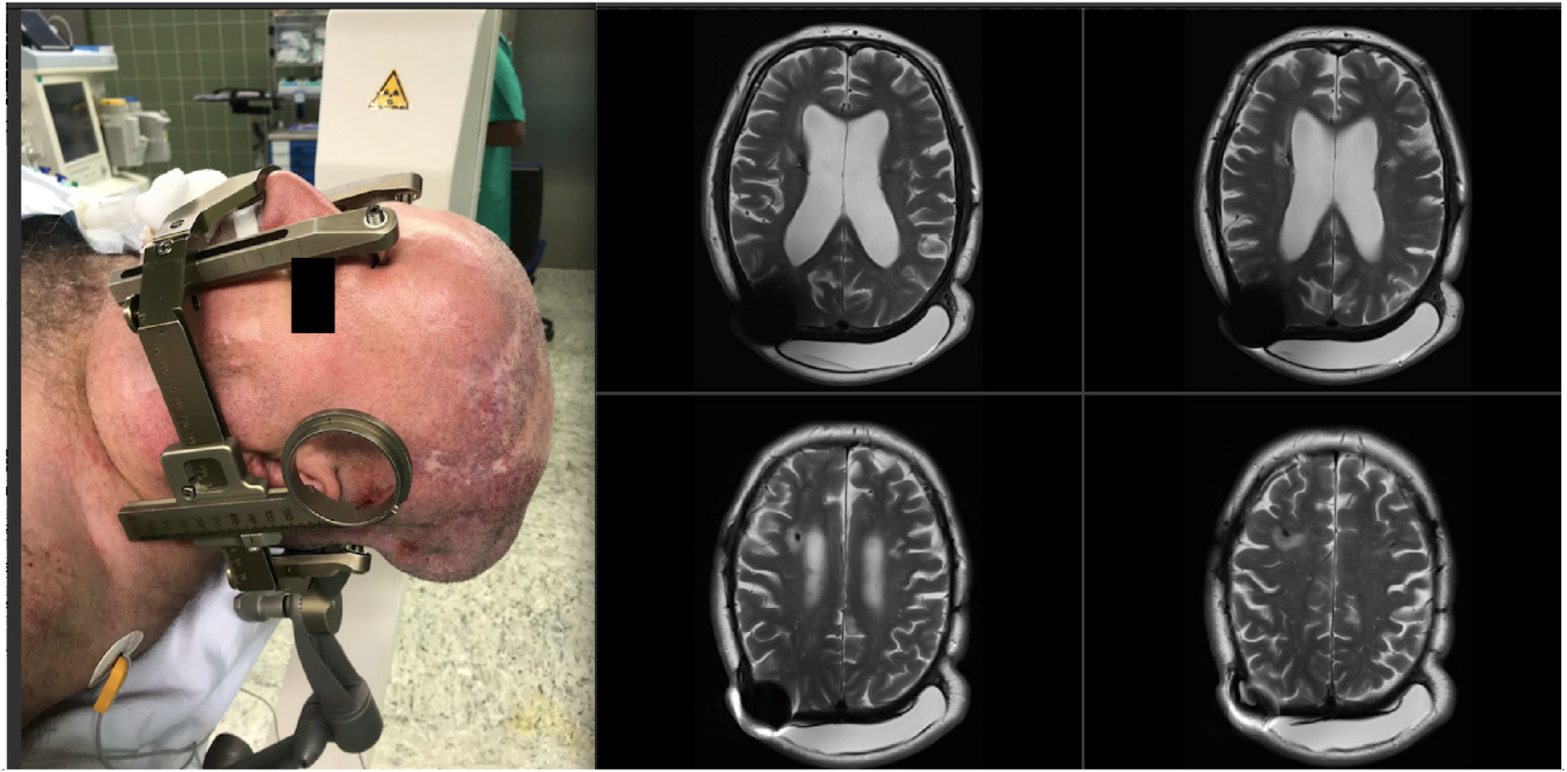
Considerations regarding TE-positioning and placement of the stereotactic frame.

At the beginning of the procedure, the Leksell coordinate frame G was placed practicing extreme caution to not puncture the TE or anchor the valve. After placement of the bilateral STN electrodes, both frontal incisions were extended to meet in the middle and mobilization was carried out. The TE was removed and a rotational-flap utilized to mobilize the gained tissue anteriorly to provide a strain-free scalp closure. Finally, the IPG was placed in a virgin paraumbilical pocket and the circuit completed with electrode extensions. Preoperative antibiotics were repeated due to the length of the procedure, which exceeded 6 ​h. Postoperative antibiotic therapy with Clindamycin and Cefuroxime was continued for another 6 days. After optimization of stimulation settings and the Parkinson medication, the patient was discharged to rehabilitative care.

## 3 Results

The relatively scanty related literature almost exclusively deals with MRI safety of TEs for breast reconstruction (Marano et al., 2017) Obtaining MRI safety data for TEs available on the German-market proved to be difficult. We contacted the manufacturer for TEs which usually provides these implants for the department of Oral- Cranio- Maxillofacial & Facial Plastic Surgery and requested information on the material used in production. The manufacturer produces two general versions of TEs differing from one another on the presence of an integrated magnet to localize the valve for staged tissue-expansion. While the TE with the permanent magnet is labelled as unsafe for MRIs, there are no MRI safety data for the TE without the permanent magnet. There can be traces-in varying quantity, of ferromagnetic material in the casing of the valve. Detailed informed patient consent in this regard was obtained keeping in mind that should the implant heat or cause distortion rendering the images not conducive to trajectory planning, a removal of the TE would need to be carried out beforehand either increasing the length of anesthesia or requiring separate procedures. We selected the crescent shaped Polytech POLYsmoooth™ 200 ​ml ​TE (without a magnet) and obtained informed-consent from the patient regarding the limited MRI-safety of the implant.

We took applicable precautions based on the literature (Marano et al., 2017) in using saline to achieve tissue expansion and monitored the patient during the 1,5 ​T MRI conducted in the usual supine position under physician supervision. Placing an additional weight over the implant was deemed unnecessary because of occipital-positioning of the TE and supine positioning of the patient. To our relief, there were no adverse events. The patient reported no heating of the implant and no discomfort. There was no valve displacement. The artifacts were unavoidable but did not hinder electrode trajectory-planning.

A general conclusion towards MRI safety of this particular implant cannot be drawn based on this one case, however. The manufacturer has since been working on a completely metal-free TE. The implant has, at the time of this publication, not been added to their product catalogue and is pending necessary approvals and testing. If the implant will be rigorously MRI tested and be labelled as MRI-safe for this specific use to make clinical decision-making easier remains to be seen. Closer partnership with the industry in communicating surgical needs in highly specialized fields like functional neurosurgery seems indispensable (Grundfest-Broniatowski, 2013)

A month after discharge, a follow-up Image with contrast medium showed contrast enhancement in the occipital scalp and along the right DBS-STN electrode in the frontal lobe. There were no laboratory or clinical findings that suggested an infection. Considering the patient's history, however, and in consensus with our department for infectious diseases, intravenous therapy with Vancomycin, Meropenem and Phosphomycin was initiated and continued in an ambulatory setting with additional implantation of a port catheter to facilitate administration. There was considerate neurological improvement under stimulation and the patient was ambulatory without the use of a wheelchair. The antibiotics were administered for another month under regular blood-sampling which was continued after the antibiotics were discontinued. A follow-up CT with contrast showed no more enhancement. The wound healing has been satisfactory after 38 months of follow-up. The laboratory parameters (C-reactive Protein and WBC Count) show no indications for a systemic infection.

## 4 Discussion and conclusion

Tissue-expansion is a time-tested technique which has, to our knowledge, not been utilized in functional neurosurgery thus far. The application however seems justified in patients with PD who have had to undergo multiple revision procedures after DBS. PD seems to affect sympathetic skin nerve-fibres, which express phosphorylated *α*-synuclein correlating with age-independent denervation of autonomic skin elements (Zange et al., 2015; Jitkritsadakul et al., 2017). The pathogenic dysfunction of *α*-synuclein associated with PD has spurred a whirl of papers in the last decade (Xu and Pu, 2016; Bengoa-Vergniory et al., 2017; Cersosimo and Benarroch, 2012; Csoti et al., 2016; Forsyth et al., 2011; Farrand et al., 2017; Reinard et al., 2016). The usefulness of coming up with a risk score for PD patients at risk for developing SDs and consequent infections and multiple revisions can be debated. Routine skin-biopsies at first DBS implantation are being considered at our department pending an ethics board review.

Regarding MRI safety, our case demonstrated the need for closer cooperation with the industry to convey the needs of highly-specialized fields. A need for MRI-safe TEs has been coming up for breast reconstruction (Marano et al., 2017; Nava et al., 2012). Incentivizing the development of such implants by increasing the number of potential users from reconstructive surgeons to include functional neurosurgeons might fuel research and development.

The provider of the TE used in our example does not publish unit prices since these are ascertained based on practice volume and geographical location. That being said, the local cost-factor should definitely be taken into account especially in lower-income areas. Hardware-free procedures like lesioning could be considered primarily or for refractory cases as worthy alternatives.

We cannot afford to undervalue meticulous planning and pluridisciplinary boards for complex cases. TEs and/or other reconstructive-procedures like rotational flaps can only be implemented in such a setting. We therefore recommend referring similar cases to high-volume centers in the best interest of the patient.

## Author statement

Nikhil Thakur, Conceptualization, Formal analysis, Investigation, Methodology, Roles/Writing - original draft, Writing - review & editing; Michael Eibach –, Investigation, Methodology; Shahram Ghanaat, Methodology; Lutz Weise, Methodology, Writing - review & editing; Volker Seifert, Supervision; Gerhard Marquardt, Supervision, Writing - review & editing; Johanna Quick-Weller; Conceptualization, Formal analysis, Investigation, Methodology, Supervision, Writing - review & editing.

The authors would like to thank Mrs. Marina Heibel for her contribution to Image 3 (TE positioning)

## Disclosures

- No financial disclosures.

- This data was presented at the following meetings:

1.) In poster format at the 18th biennial meeting of the World Society for Stereotactic and Functional Neurosurgery in New York City in June 2019.

2.) In e-poster format at the EANS annual meeting in Dublin in September 2019 and.

3.) As an operative technique oral presentation during the “Pain Operative Techniques and Case-based Discussion” Session at the 2019 Congress of Neurological Surgeons’ annual meeting, October 19–23, in San Francisco.

## Author's statements

- I take full responsibility for the data, the analysis and interpretation, and the conduct of the research; I have full access to all of the data.

- I did not receive any financial support in regards to this publication.

- I have the express permission of the patient to use his data as an example of the discussed technique. The signed consent form in German language – the patient's mother tongue, is on file – should this be required by the editor(s)*.*
